# Mucin-hypersecreting bile duct neoplasm characterized by clinicopathological resemblance to intraductal papillary mucinous neoplasm (IPMN) of the pancreas

**DOI:** 10.1186/1477-7819-5-98

**Published:** 2007-08-28

**Authors:** Yo-ichi Yamashita, Kengo Fukuzawa, Akinobu Taketomi, Shinichi Aishima, Tomoharu Yoshizumi, Hideaki Uchiyama, Eiji Tsujita, Norifumi Harimoto, Noboru Harada, Kenzo Wakasugi, Yoshihiko Maehara

**Affiliations:** 1 Department of Surgery and Science, Graduate School of Medical Sciences, Kyushu University, 3-1-1 Maidashi, Higashi-ku, Fukuoka 812-8582, Japan; 2 Department of Surgery, Oita Red Cross Hospital, 3-2-37 Chiyo-machi, Oita 870-0033, Japan; 3 Department of Anatomic Pathology, Graduate School of Medical Sciences, Kyushu University, 3-1-1 Maidashi, Higashi-ku, Fukuoka 812-8582, Japan

## Abstract

**Background:**

Although intraductal papillary mucinous neoplasm (IPMN) of the pancreas is acceptable as a distinct disease entity, the concept of mucin-secreting biliary tumors has not been fully established.

**Case presentation:**

We describe herein a case of mucin secreting biliary neoplasm. Imaging revealed a cystic lesion 2 cm in diameter at the left lateral segment of the liver. Duodenal endoscopy revealed mucin secretion through an enlarged papilla of Vater. On the cholangiogram, the cystic lesion communicated with bile duct, and large filling defects caused by mucin were observed in the dilated common bile duct. This lesion was diagnosed as a mucin-secreting bile duct tumor. Left and caudate lobectomy of the liver with extrahepatic bile duct resection and reconstruction was performed according to the possibility of the tumor's malignant behavior. Histological examination of the specimen revealed biliary cystic wall was covered by micropapillary neoplastic epithelium with mucin secretion lacking stromal invasion nor ovarian-like stroma. The patient has remained well with no evidence of recurrence for 38 months since her operation.

**Conclusion:**

It is only recently that the term "intraductal papillary mucinous neoplasm (IPMN)," which is accepted as a distinct disease entity of the pancreas, has begun to be used for mucin-secreting bile duct tumor. This case also seemed to be intraductal papillary neoplasm with prominent cystic dilatation of the bile duct.

## Background

Some cases of papillary adenocarcinoma, cystadenocarcinoma, cystadenoma, or papillomatosis have been described as mucin-secreting biliary tumors [1-3]. Although intraductal papillary mucinous neoplasm (IPMN) of the pancreas is acceptable as a distinct disease entity, [4-6] the concept of mucin-secreting biliary tumors has not been established [7,8]. Kim *et al*., first used the term "IPMN" for nine cases of mucin-hypersecreting bile duct tumor in 2000 [9], and the condition has been described with increasing frequency in recent years [7,10-12]. We herein report a case of mucin-secreting biliary neoplasm that is clinicopathologically similar to IPMN of the pancreas.

## Case presentation

A 71-year-old Japanese woman was referred to our hospital for evaluation of her elevation of serum gamma glutamyl transferase levels to 135 IU/L (reference value 16–73 IU/L), which were incidentally found in a biochemical test in medical health check. She had no symptoms of epigastralgia or jaundice. Her physical examination and past medical history was unremarkable. She did not smoke or drink. The remainder of her serum chemistries, complete blood count, and coagulation profile were all normal. Serum levels of tumor marker such as carcinoembryonic antigen (CEA) and carbohydrate antigen 19-9 (CA19-9) were within normal limits.

**Figure 1:**
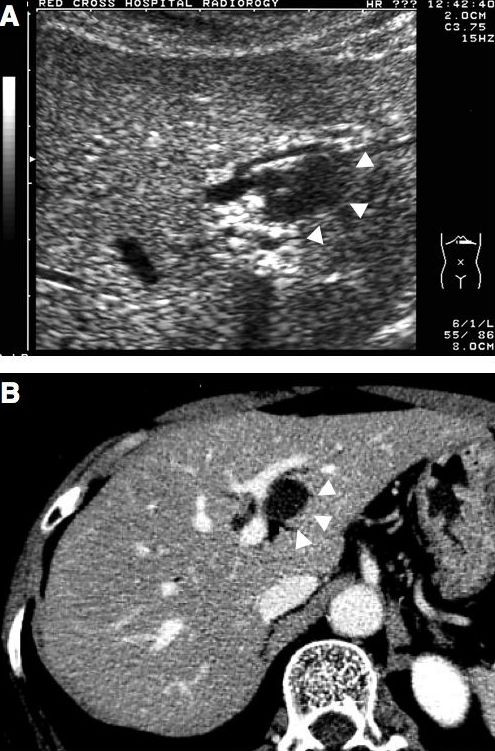
Abdominal ultrasonography (A) and computed tomography (B) show a cystic lesion measuring 2.0 cm in maximal diameter at the left lateral segment of the liver with peripheral left lateral anterior subsegmental bile duct (B3) dilatation. Arrows head indicate the cystic lesion.

**Figure 2:**
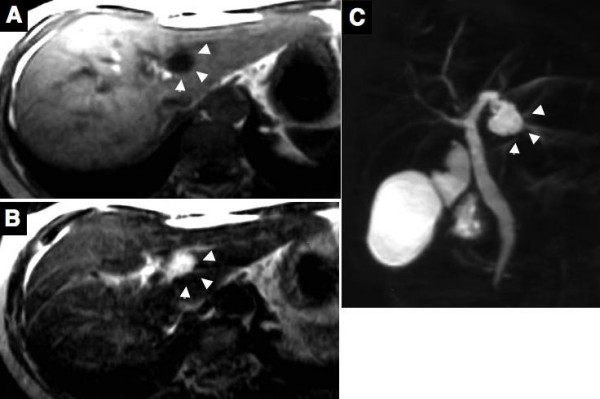
Magnetic resonance imaging (MRI) reveals the cystic lesion as low in the T1-weighted image (A) and as high in the T2-weighted image (B). MR cholangiography shows a cystic lesion at the left lobe of the liver, but a filling defect in the bile duct and a communication between the cystic lesion and bile duct could not be defined (C). Arrows head indicate the cystic lesion.

**Figure 3:**
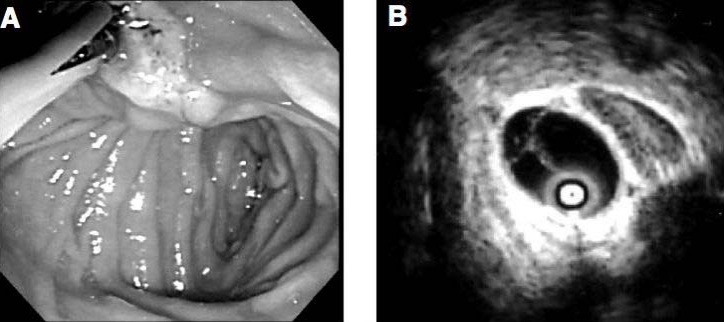
Endoscopic image of the duodenum shows mucin draining from a patulous papillary orifice (A). Endoscopic ultrasonography showed no mass protruding into the lumen in the bile duct and the cystic lesion at the left lateral segment of the liver (B).

**Figure 4:**
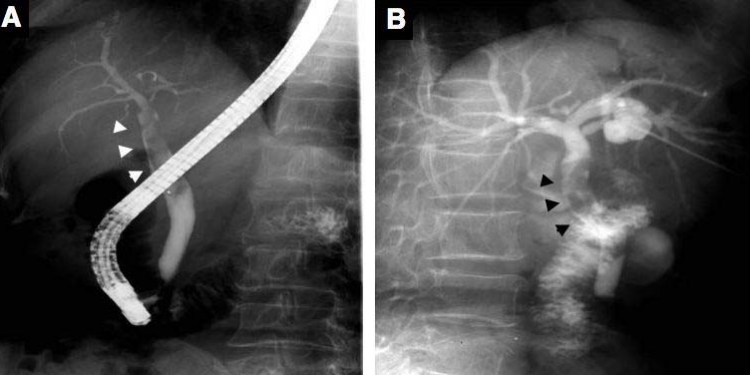
Endoscopic retrograde cholangiography (A) shows a dilated common bile duct with defined filling defects corresponding to mucin. Percutaneous transhepatic cholangiography (B) also shows mucin in the common bile duct and a communication between the cystic lesion and bile duct. However, the filling defect corresponding to the tumor component in the cystic lesion could not be defined. Arrows head indicate mucin in the common bile duct.

Abdominal ultrasonography (US) and computed tomography (CT) demonstrated a cystic lesion measuring 2.0 cm in maximal diameter at the left lateral segment of the liver with peripheral left lateral anterior subsegmental bile duct (B3) dilation (Figure 1A, and 1B). Magnetic resonance (MR) imaging also confirmed a cystic lesion at the left lateral segment of the liver, but the presence of a mural nodule in the cystic lesion or mucin was not confirmed (Figure 2A,2B). MR cholangiography showed a cystic lesion at the left lobe of the liver, but a filling defect in the bile duct and a communication between the cystic lesion and bile duct could not be defined (Figure 2C). Duodenoscopy showed a widely patent papillary orifice with extruded mucoid material, and endoscopic ultrasonography (EUS) could not detect a tumor component in the bile duct or cystic lesion (Figure 3A,3B). Endoscopic retrograde cholangiogram (ERC) demonstrated amorphous filling defects in the common bile duct corresponding to mucin, and percutaneous transhepatic cholangiograms (PTC) revealed communication between the hepatic cyst and the major bile duct (Figure 4A,4B). The cytological examinations through both ERC and PTC routes were negative for malignancy.

**Figure 5:**
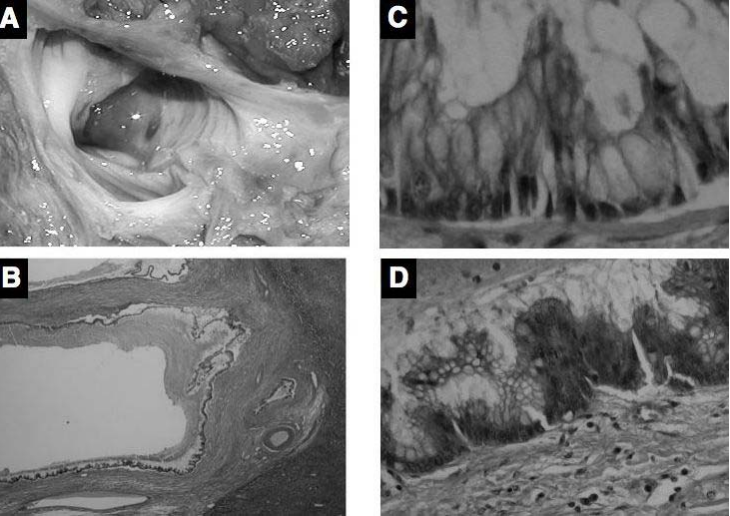
(A) The gross appearance of the resected specimen does not show a mass protruding into lumen in the cystic lesion with mucin. (B) Microscopically, the cystically dilated bile duct does not have a mass of protruding lesion composed of papillary growth with fibrovascular cores or villous structures (hematoxylin and eosin ×40). (C) Cyst wall was lined by tall columnar epithelium with mucin hypersecretion (Hematoxylin and eosin ×200). (D) Neoplastic cells with hyperchromatic nuclei and loss of cell polarity was occasionally observed, but stromal invasion was not present (Hematoxylin and eosin ×200).

Left and caudate lobectomy of the liver with extrahepatic bile duct resection and reconstruction was performed because the lesion seemed to be a potential malignancy. Gross examination of the resected specimens revealed no obvious mass protruding into the lumen, and only a markedly dilated bile duct was observed at a glance (Figure 5A). Microscopically, the cystically dilated bile duct was lined by tall columnar epithelium with micropapillary features and mucin hypersecretion, but the tall papillary growth with fibrovascular cores or villous structures showing mass component was not present (Figure 5B,5C). These neoplastic cells with hyperchromatic nuclei and loss of cell polarity, lacking stromal invasion was observed (Figure 5D). Ovarian-like stroma typically seen in hepatobiliary cystadenomas was not observed in the wall. On immunohistochemistry using standard streptavidin-biotin-peroxidase method, meoplastic cells were positive for MUC2 and MUC5AC, but negative for MUC1, CK20. The patient has been well without any evidence of recurrence for 38 months since her operation.

## Discussion

While the concept of mucin-producing pancreatic tumors is well-recognized, the concept of a mucin-secreting biliary tumor has not been well-established. Considering the shared embryonic origin of the two duct system [13], mucin-secreting bile duct tumor, like an IPMN of the pancreas, may theoretically also exist. Based on the imaging for intraductal papillary tumors of the bile duct, mucin component was detected as filling defect at cholangiography and the papillary tumor was detected as polypoid mass in the dilated biliary tree as well as IPMN of pancreas [1,12]. Zen *et al*., summarized pathological similarities and differences between IPN of bile duct and IPMN of pancreas [8]. Similar findings were intraductal papillary proliferation with fibrovascular core, occasional mucin hypersecretion, most of mucin profiles, and patient outcome, while dissimilar findings were pointed out to be the incidence of immunohistochemical cytokeratin 20 expression, the percentage of gastric-type tumors, and the frequency of mucin hypersecretion. IPMN of the pancreas is a grossly visible, non-invasive, mucin-producing, predominantly papillary epithelial neoplasm with varying degree of cellular atypia, arising from pancreastic ducts with duct dilatation [6]. A mass component of papillary proliferation commonly seen in IPMN of pancreas was absent and micropapillary proliferation was predominant in our case, but the histological features of cystic dilated duct with mucin hypersecreation, and moderate cellular atypia were resemble with that of IPMN of pancreas. On the other hand, hepatobiliary cystadenoma/adenocarcinoma in middle-aged women have been reported to have ovarian-like stroma [14,15] and a large number of these cases would enter the disease entity of a mucinous cystic neoplasm (MCN) of the pancreas [16]. Our case did not have ovarian-like stroma in the cystic wall. These findings and mucin profiles suggest that our biliary lesion resemble with cystic variant of intraductal papillary neoplasm of the bile duct, proposed by Zen *et al*., [17]. Lim JH., [12] have summarized the characteristics observed in the imaging of IPMN of the bile duct from 15 cases, and our case is compatible with these descriptions at an aneurysmal type. Also in our case, ERC or PTC instead of US, CT, or MRI made it possible to detect mucin. We therefore consider that ERC or PTC is necessary to diagnose IPMN of the bile duct.

According to the adenoma-carcinoma sequences in the IPMN of the pancreas [18] we treated our case according to the malignant behavior. Percutaneous transhepatic cholangioscopy (PTCS) was recommended for the accurate diagnosis of IPMN of the bile duct, [1,9,11] but we did not perform PTCS because of its invasiveness, the time required to complete the procedure, and the risk of malignant seeding of the tract [1]. Lim JH *et al*., [12] have also reported that 7 of 15 (47.3%) patients with IPMN of the bile duct had multiple IPMN in their resected specimens. Therefore, intraoperative cholangioscopy and frozen histology should have been performed in our case. If frozen histology had revealed no malignancy in this case, more limited resection such as left lobectomy of the liver without caudate lobectomy of the liver and extrahepatic bile duct resection could have been performed.

If more cases of IPN of the bile duct are reported, consensus could be achieved regarding the optimal and least invasive preoperative evaluation and treatment plans such as indications for surgery and the extent of resection.

## Conclusion

It is only recently that the term "intraductal papillary mucinous neoplasm (IPMN)," which is accepted as a distinct disease entity of the pancreas, has begun to be used as "intraductal papillary neoplasm of the bile duct (IPN-B)" for mucin-secreting bile duct tumor. If more cases of IPN of the bile duct are reported, consensus could be achieved regarding the optimal and least invasive preoperative evaluation and treatment plans.

## Competing interests

The author(s) declare that they have no competing interests.

## Authors' contributions

YIY, KF, and KW participated in the patient's care and drafting of manuscript. AT, TY, HU, NH, ET, NH, and YM participated in collecting the patient's data and drafting the manuscript. YIY and SA participated in the pathological evaluation. All authors read and approved the final manuscript.

## References

[B1] Sakamoto E, Hayakawa N, Kamiya J, Kondo S, Nagino M, Kanai M, Miyachi M, Uesaka K, Nimura Y (1999). Treatment strategy for mucin-producing intrahepatic cholangiocarcinoma: Value of percutaneous transhepatic biliary drainage and cholangioscopy. World J Surg.

[B2] Styne P, Warren GH, Kumpe DA, Halgrimson C, Kern F (1986). Obstructive cholangitis secondary to mucus secreted by a solitary papillary bile duct tumor. Gastroenterology.

[B3] Wang YL, Lee SD, Lai KH, Wang SS, Lo KJ (1993). Primary biliary cystic tumors of the liver. Am J Gastroenterol.

[B4] Fukushima N, Mukai K (1999). Pancreatic neoplasms with abundant mucus production: emphasis on intraductal papillary-mucinous tumors and mucinous cystic tumors. Adv Anat Pathol.

[B5] Longnecker DS, Hruban RH, Adler G, Kloppel G (2000). Intraductal papillary-mucinous neoplasm of the pancreas; World Health Organization International Histological Classification of Tumors, Pathology and Genetics of Tumours of the Digestive System.

[B6] Hruban RH, Takaori K, Klimstra DS, Adsay NV, Albores-Saaverdra J, Biankin AV, Biankin SA, Compton C, Fukushima N, Furukawa T, Goggins M, Kato Y, Kloppel G, Longnecker DS, Luttges J, Maitra A, Offerhaus GJ, Shimizu M, Yonezawa S (2004). An illustrated consensus on the classification of pancreatic intraepithelial neoplasia and intraductal papillary mucinous neoplasms. Am J Surg Pathol.

[B7] Shibahara H, Tamada S, Goto M, Oda K, Nagino M, Nagasaka T, Batra SK, Hollingsworth MA, Imai K, Nimura Y, Yonezawa S (2004). Pathologic features of mucin-producing bile duct tumors: two histopathologic categories as counterparts of pancreatic intraductal papillary-mucinous neoplasms. Am J Surg Pathol.

[B8] Zen Y, Fujii T, Itatsu K, Nakamura K, Minato H, Kasashima S, Kurumaya H, Katayanagi K, Kawashima A, Masuda S, Niwa H, Mitsui T, Asada Y, Miura S, Ohta T, Nakanuma Y (2006). Biliary papillary tumors share pathological features with intraductal papillary mucinous neoplasm of the pancreas. Hepatology.

[B9] Kim HJ, Kim MH, Lee SK, Yoo KS, Park ET, Lim BC, Park HJ, Myung SJ, Seo DW, Min YI (2000). Mucin-hypersecreting bile duct tumor characterized by a striking homology with an intraductal papillary mucinous tumor (IPMT) of the pancreas. Endoscopy.

[B10] Oshikiri T, Kashimura N, Katanuma A, Maguchi H, Shinohara T, Shimizu M, Kondo S, Katoh H (2002). Mucin-secreting bile duct adenoma-clinicopatological resemblance to intraductal papillary mucinous tumor of the pancreas. Dig Surg.

[B11] Somogri L, Dimashkieh H, Weber FL, Buell J (2003). Biliary intraductal papillary mucinous tumor: diagnosis and localization by endoscopic retrograde cholangioscopy. Gastrointest Endosc.

[B12] Lim JH, Yoo KH, Kim SH, Kim HY, Lim HK, Song SY, Nam KJ (2004). Intraductal papillary mucinous tumor of the bile ducts. RadioGraphics.

[B13] Sadler TW (2006). Langman's Medical Embryology.

[B14] Buetow PC, Buck JL, Pantongrag-Brown L, Ros PR, Devaney K, Goodman ZD, Cruess DF (1995). Biliary cystadenoma and cystadenocarcinoma: clinical-imaging-pathologic correlations with emphasis on the importance of ovarian stroma. Radiology.

[B15] Devaney K, Goodman ZD, Ishak KG (1994). Hepatobiliary cystadenoma and cystadenocarcinoma. A light microscopic and immunohistochemical study of 70 patients. Am J Surg Pathol.

[B16] Inagaki M, Maguchi M, Kino S (1999). Mucin-producing tumors of the pancreas: clinicopathological features, surgical treatment, and outcome. J Hepatobiliary Pancreat Surg.

[B17] Zen Y, Fujii T, Itatsu K, Nakamura K, Konishi F, Masuda S, Mitsui T, Asada Y, Miura S, Miyayama S, Uehara T, Katsuyama T, Ohta T, Minato H, Nakanuma Y (2006). Biliary cystic tumors with bile duct communication: a cystic variant of intraductal papillary neoplasm of the bile duct. Mod Pathol.

[B18] Obara T, Maguchi H, Saitoh Y (1993). Mucin-producing tumor of the pancreas: Natural history and serial pancreatogram changes. Am J Gastroenterol.

